# Deep venous thrombosis and hyponatremia associated with citalopram use for behavioral symptoms in Parkinson’s disease: a case report

**DOI:** 10.1186/s12877-023-04057-z

**Published:** 2023-06-01

**Authors:** Afaf Albalawi

**Affiliations:** Department of Internal Medicine, University of Tabuk, B.O.Box:4279, Tabuk City, 71491 Tabuk Saudi Arabia

**Keywords:** Deep venous thrombosis, Hyponatremia, Citalopram, Parkinson’s disease dementia

## Abstract

**Background:**

Evidence is limited regarding the optimal therapeutic approach for neuropsychiatric symptoms associated with Parkinson’s disease dementia (PDD). Selective serotonin reuptake inhibitors (SSRIs) are widely used for mood disorders and behavioral symptoms in older adults with cognitive impairment, but they have limited efficacy in patients with PDD. The effect of SSRIs on hemostasis is also unclear. This report describes a patient with PDD who developed deep venous thrombosis (DVT) and hyponatremia after initiating citalopram treatment.

**Case presentation:**

An 86-year-old woman with PDD presented to our emergency department with altered mental status, generalized weakness, and left lower leg swelling. Citalopram was begun 4 weeks previously for behavioral changes and was discontinued 2 days before presentation because of excessive fatigue. At presentation, her plasma sodium level was 123 mg/dL. Brain computed tomography showed age-related changes. Doppler ultrasound revealed a DVT in the left lower leg. The patient was treated with hypertonic saline and intravenous heparin. After normalization of her sodium, she was discharged on donepezil and apixaban. At follow-up, her sodium remained normal, and her cognition and behavior were noticeably improved.

**Conclusion:**

Older adults with Parkinson’s disease are sensitive to adverse effects of psychotropic agents, including SSRIs, which are not recommended first-line agents for behavioral symptoms in PDD. Upon initiating SSRIs in older patients with functional decline and multiple comorbidities, physicians need to evaluate the patient’s risk factors for bleeding or thrombosis. Physical activities should also be maintained as much as possible.

## Background

High-level evidence is lacking regarding the optimal therapeutic management of neuropsychiatric symptoms in patients with Parkinson’s disease dementia (PDD) or Lewy body dementia (LBD) [1]. Despite reports of limited efficacy, selective serotonin reuptake inhibitors (SSRIs) are widely used for neuropsychiatric symptoms in patients with these disorders [2]. SSRIs can worsen cognitive and motor symptoms associated with Lewy body dementia [3].

Evidence regarding the effects of SSRIs on vascular hemostasis is contradictory and inconsistent. This case report describes the development of deep venous thrombosis (DVT) and hyponatremia in a patient treated with citalopram (an SSRI) for behavioral symptoms related to PDD. Other case reports have also hypothesized a connection between SSRIs and VTE which warrant further study [4, 5].

## Case presentation

An 86-year-old woman with Parkinson’s disease, hypertension, diabetes mellitus, hyperlipidemia, and osteoporosis presented to our emergency department with altered mental status, reduced oral intake, generalized weakness, and left lower leg swelling. At baseline, she was functional, as evidenced by dependence for both basic and instrumental activities of daily living, and cognitively impaired. She had no history of venous thromboembolism, bleeding, or psychiatric illness. An echocardiogram 5 months previously was normal except for grade 1 diastolic dysfunction. Her current medications included gliclazide, metformin, valsartan, and atorvastatin; she was receiving no Parkinson’s disease treatment. Citalopram was begun 4 weeks previously for behavioral changes (agitation, hallucinations, repetitive vocalizations, and insomnia). The initial dose of 10 mg daily was ineffective, and the patient became restless. Against medical advice, the dose was increased to 20 mg daily after only 1 week. Two days before presentation, citalopram was discontinued because of excessive fatigue.

At presentation, the patient was disoriented and lethargic. Her blood pressure was 104/51 mmHg, random blood glucose was 125 mg/dL, and plasma sodium was 123 mg/dL. Her plasma sodium was normal prior to the initiation of citalopram. Other blood tests (including adrenal function studies) were normal, and there was no evidence of malignancy or an autoimmune disease. Brain computed tomography showed age-related changes. Doppler ultrasound revealed a DVT in the left lower leg.

The patient was admitted to the hospital and treated with hypertonic saline and intravenous heparin. Her chronic medications were placed on hold upon admission. After 2 days, her sodium level normalized. The patient was discharged on donepezil 5 mg OD and apixaban 2.5 mg BID. Other chronic medications, excluding gliclazide and citalopram (which had been discontinued 2 days prior to admission), were resumed.

At 4 weeks follow-up, her plasma sodium remained normal, and her cognitive function and behavior were noticeably improved.

## Discussion

SSRIs may alleviate dementia-related behavioral symptoms [6, 7], such as agitation, but little evidence supports their use for PDD. Multiple studies have reported limited effectiveness of SSRIs for neuropsychiatric symptoms related to PDD [8, 9]. In our case, citalopram was not helpful and led to worsened cognition, as well as serious adverse events (hyponatremia and DVT) requiring hospitalization within 4 weeks of initiation.

In addition to the unclear role of SSRIs in PDD and LBD, evidence regarding the effects of SSRIs on hemostasis is also contradictory. Some studies reported that SSRIs increased the risk of bleeding [10, 11], whereas others reported an increased risk of thrombosis and venous thromboembolism (VTE) [12–14]. A systematic review with meta-analysis concluded that SSRIs increase VTE risk, but more research is required regarding the causative mechanism(s) [14]. Another systematic review reported that SSRIs can cause VTE either directly (platelet aggregation, venous stasis) or indirectly (obesity, sedation) [15]. Other evidence suggests that the effects on platelets vary over time, with an initial increased tendency for thrombosis and a later increased risk of bleeding upon repeated dosing [4]. We hypothesize that our patient’s DVT was due to the effects of hyponatremia related to the syndrome of inappropriate antidiuretic hormone secretion (SIADH) [16, 17], which led to hypoactive delirium, bed confinement, hypercoagulability, and eventual DVT.

The Naranjo scale was developed to help standardize assessment of causality for adverse drug reactions [18]. Our patient has a single alternative cause for DVT which is functional decline, with no other risk factor prior to Citalopram initiation. She scored 5 on the Naranjo scale, suggesting that this adverse event was probably related to citalopram induced SIADH which then complicated with DVT.

Emerging evidence suggests that dopaminergic agonists are beneficial for nonmotor symptoms of PDD, including depression, and that antidepressants acting on both the serotonin and noradrenaline systems reduce depression [19]. Cholinesterase inhibitors could improve cognitive function with different side effects and potential to worsen the motor symptoms associated with PDD and LBD [2, 20]. In a randomized clinical trial, citalopram was discontinued in 71% of patients with LBD because of neurologic side effects and worsened psychiatric symptoms [3]. Despite the possible benefits of dopaminergic agonists and cholinesterase inhibitors for behavioral symptoms associated with dementia, adverse events (orthostatic hypotension, hallucinations, and impulse control disorders for dopaminergic agonists; nausea, vomiting, diarrhea, and insomnia for cholinesterase inhibitors) and withdrawal symptoms with abrupt discontinuation should be discussed with patients and caregivers.

Prior studies have established an association between SSRIs and SIADH [8, 21], and older adults are especially vulnerable to SSRI-related electrolyte disturbances. The patient in this case report had normal sodium levels prior to citalopram treatment, and they decreased after citalopram treatment began. Close monitoring of sodium levels is crucial when treating older adults with SSRIs, especially when SSRIs are combined with other medications that may affect sodium levels, such as sulfonylureas (e.g., gliclazide), to avoid synergistic effects and the serious consequences of hyponatremia.

This case highlights the uncertainty, limited efficacy, and reduced tolerability of SSRIs when used for neuropsychiatric symptoms related to PDD. First-line therapy for these symptoms should focus on non-pharmacologic measures, followed by dopaminergic agonists (which also manage the motor symptoms of Parkinson’s disease) or cholinesterase inhibitors. When initiating SSRIs in older patients with functional decline and multiple comorbidities, the risk factors for thrombosis or bleeding need to be evaluated. Mobility should be maintained as much as possible.

## Conclusions

SSRIs can cause life-threatening adverse events when used for managing behavioral symptoms associated with PDD and LBD. Initial management should focus on non-pharmacologic interventions. Further research is needed to guide therapy for neuropsychiatric symptoms in patients with PDD or LBD.

This case report highlights VTE associated with citalopram-induced SIADH, a potentially life-threating adverse event for a commonly prescribed medication.

## Data Availability

Not applicable.

## Abbreviations

PDD: Parkinson’s disease dementia
SSRIs: Selective serotonin reuptake inhibitors
DVT: Deep venous thrombosis
LBD: Lewy body dementia
VTE: Venous thromboembolism
SIADH: Syndrome of inappropriate antidiuretic hormone secretion
